# Professionals’ Perceptions on Implementing an Adapted Lifestyle Coaching Program for People with Physical Disabilities

**DOI:** 10.3390/healthcare13161978

**Published:** 2025-08-12

**Authors:** Elizabeth H. Douma, Trynke Hoekstra, Jesse K. Nijboer, Martin Fluit, Lieneke Vos, Femke Hoekstra

**Affiliations:** 1 Department of Health Sciences, Vrije Universiteit Amsterdam and Amsterdam Public Health Research Institute, 1081 HV Amsterdam, The Netherlandstrynke.hoekstra@vu.nl (T.H.);; 2 Stichting Special Heroes, 3584 AA Utrecht, The Netherlands; mfluit@specialheroes.nl (M.F.);; 3 Department of Medicine, Division of Social Medicine, The University of British Columbia, Vancouver, BC V5Z 1M9, Canada; 4 Centre for Chronic Disease Prevention and Management, The University of British Columbia, Kelowna, BC V1V 1V7, Canada

**Keywords:** health promotion, rehabilitation, coaching program, interviews

## Abstract

**Background/Objectives:** Evidence-based lifestyle coaching programs have been developed to support people with disabilities in adopting healthy behaviors, and to ultimately contribute to enhancing their overall well-being. However, when implementing such programs in new settings, adaptations may be needed to ensure a successful implementation process. This study aimed to explore professionals’ perceptions on an adapted evidence-informed lifestyle coaching program (Healthy Habits Coaching) for people with physical disabilities to inform the implementation of the program in Dutch rehabilitation and/or community settings. **Methods:** A qualitative study with semi-structured interviews was conducted. The study was performed from a pragmatic perspective using an integrated knowledge translation approach. Ten professionals who had experience with offering, delivering, and/or implementing lifestyle coaching programs were enrolled. Interview questions focused on participants’ perceptions on implementing the Healthy Habits Coaching in Dutch settings. A directed content analysis was used to analyze the data. **Results:** Participants highlighted the importance of implementing lifestyle coaching tailored to people with physical disabilities. While participants were generally positive about the implementation of Healthy Habits Coaching, they had mixed opinions on its added value alongside existing lifestyle programs and on the core components, particularly the free coaching model and the use of volunteer coaches with lived experience. Participants underlined that for a successful adoption and implementation, the added value, (scientific) foundation, financial basis, and organizational structure of the program should be clearly communicated. **Conclusions:** The findings provide directions for how, where, and by whom an adapted lifestyle program (Healthy Habits Coaching) for people with physical disabilities could be implemented in Dutch rehabilitation and community settings. This study demonstrates an example of how an evidence-based lifestyle program can be prepared for implementation in a new setting, presenting an efficient and promising strategy to enhance overall well-being among people with disabilities.

## 1. Introduction

Engaging in healthy lifestyle behaviors, such as regular physical activity, eating a healthy diet, obtaining sufficient sleep, and avoiding tobacco and alcohol use, can improve physical and mental well-being in people with disabilities [1,2,3,4,5,6]. Despite these well-documented benefits, people living with physical disabilities (PLWDs) show generally lower levels of health-promoting behaviors compared to the general population due to a variety of unique barriers PLWDs experience in accessing services and engaging in health-promoting behaviors. To illustrate, barriers to participating in physical activity among PLWDs have been reported across multiple levels, including intrapersonal (e.g., pain and fatigue), interpersonal (e.g., limited social support), institutional (e.g., inaccessible facilities), community (e.g., lack of adapted equipment), and policy (e.g., insufficient funding and transportation limitations) [1]. Many of the reported barriers, such as inaccessibility of environments and the lack of knowledge or skills among health professionals and coaches to support PLWDs, are particularly unique to this population, contributing to their disproportionate exclusion from health promotion efforts. This disparity highlights the importance of supporting PLWDs in adopting and maintaining a healthy lifestyle.

Health behavior programs including tailored lifestyle coaching can be an effective approach to promote healthy behaviors, such as physical activity and healthy eating [7,8,9,10]. Transferring existing, successful lifestyle behavioral programs tailored to PLWDs to new settings is important to enhance their impact and ensure that more people around the world can benefit from them. Instead of ‘re-inventing the wheel’, using existing knowledge and best practices provides a practical and efficient strategy for addressing health disparities in PLWDs and contributing to enhancing overall well-being in this population. However, when transferring existing programs to new settings, adaptations may be needed to support a successful adoption and implementation process.

Adaptations of evidence-based health programs can be defined as ‘a systematically planned and proactive process of intervention modification with the aim to suit the specific characteristics and needs of a new context and enhance intervention acceptability’ [11]. Movsisyan et al. [11] conducted a systematic review of guidance papers that address the adaptation of health programs to new contexts. The authors distinguished four phases of this process: the exploration phase, the preparation phase, the implementation phase, and the sustainability phase. The exploration phase focuses on selecting and exploring the program (e.g., acquiring the original program material). The preparation phase includes identifying potential mismatches, developing an intervention model, and establishing networks and capacity (e.g., stakeholder engagement). The implementation phase involves modifications and pilot testing the adapted program. It is crucial to ensure that the program’s core components, identified as the main drivers of effectiveness, remain unchanged during these modifications. Finally, the sustainment phase focuses on implementation and maintenance of the program in the new context.

An example of an evidence-based behavioral program that is ready for implementation in other settings to promote a healthy lifestyle among people with disabilities is Get in Motion (GiM). GiM is a Canadian, telephone-based coaching service for PLWDs. In this program, where participation is free for clients, volunteers are trained to coach PLWDs to increase their leisure-time physical activity [12]. GiM participants have shown to indeed have increased and subsequently sustained their physical activity levels [13]. Due to its telephone-based nature, the program is low in cost and has the potential to serve many clients across a geographically large area.

At the beginning of the COVID-19 pandemic, GiM was re-launched to support Canadians with physical disabilities in becoming and/or staying active at home. Via international collaborations and exchange, the re-launch of GiM was picked up by Stichting Special Heroes (https://specialheroes.nl/), a Dutch non-profit community organization that promotes an active and healthy lifestyle in people with disabilities via a variety of activities and programs. With financial support from the Dutch Ministry of Health, Welfare, and Sport, Stichting Special Heroes initiated the transfer of GiM to the Dutch context. As part of the exploration and preparation phase [11], Stichting Special Heroes organized various meetings with stakeholders (e.g., counselors, researchers, and people with lived experience) to inform modifications of the Canadian GiM program to improve its fit with the Dutch context. For example, it was decided to change the name to Healthy Habits Coaching and expand the focus beyond physical activity to include sleep and nutrition. This aligns with the organizational mission of Special Heroes and the shift in the Dutch rehabilitation system towards promoting a healthy lifestyle rather than solely physical activity. Rehabilitation care refers here to specialized medical services aimed at improving the health and functional abilities of people with disabilities and/or chronic conditions (e.g., brain disorders, chronic pain, or spinal cord injury). It focuses on preventing and managing long-term physical impairments through a multidisciplinary approach, involving various healthcare professionals. In the Netherlands, this care is provided in settings like hospitals or rehabilitation centers and can be delivered as inpatient, outpatient, or consultation-based services.) Table 1 provides an overview of the key aspects of GiM and Healthy Habits Coaching, including key modifications.

The goal of Special Heroes is to implement Healthy Habits Coaching into various rehabilitation and/or community settings across the Netherlands to provide lifestyle support to different groups of Dutch adults with physical disabilities and/or chronic diseases. However, before starting this implementation process it is important to more deeply understand how research users perceive the adapted Healthy Habits Coaching program. Furthermore, input and guidance from research users are needed to inform the implementation and long-term sustainability of the Healthy Habits Coaching program in the Netherlands. To the best of our knowledge, no studies have systematically explored the preparation of the implementation of adapted, evidence-based lifestyle coaching programs for PLWDs, such as GiM, in non-English-speaking countries, highlighting a significant gap in the literature. This study addresses this gap by exploring professionals’ perceptions on an adapted evidence-informed lifestyle coaching program (i.e., Healthy Habits Coaching) for PLWDs in order to inform the implementation of the program in rehabilitation and/or community settings in the Netherlands. This paper specifically targets the preparation phase of the Movsisyan et al. [11] adaptation process.

## 2. Materials and Methods

### 2.1. Project Overview

A qualitative study with semi-structured, in-depth interviews was conducted to explore professionals’ perceptions. Using an Integrated Knowledge Translation (IKT) approach, this study was designed and conducted in partnership with representatives (MF, LV, and DV) of the community organization Stichting Special Heroes. IKT was defined here as ‘the meaningful engagement of the right research users at the right time throughout the research process’ [14,15]. Researchers (EHD, FH, TH, and JKN) and research users (MF, LV, and DV) worked collaboratively together throughout the research process to enhance the study’s relevance and impact [14,15]. In line with an IKT approach, the study was performed from a pragmatic perspective, in which research questions were based on practical, real-world issues encountered, and the findings were used to produce useful knowledge by answering these questions [16,17]. As such, decisions in the process of this study were made based on practical relevance [16]. Pragmatism follows an ontological relativist paradigm, indicating that reality is related to a certain situation and created by individuals. In other words, truth is made by one’s experiences [17].

This study was not subject to the Medical Research Involving Human Subjects Act (WMO), but ethical and privacy-related considerations were checked through the review process of the ethical committee of the Faculty of Science of the Vrije Universiteit Amsterdam (Bethcie; https://vu.nl/en/about-vu/faculties/faculty-of-science/more-about/research-ethics-review-committee-beta, accessed on 5 August 2025). Informed consent was obtained from all participants prior to the interviews. Throughout the study, peer debriefing within the research team and an audit trail were conducted to enhance confirmability [18].

### 2.2. Study Population and Recruitment

Participants were recruited through purposive sampling [15]. Two groups of professionals were included: lifestyle coaches and implementation experts. Inclusion criteria for lifestyle coaches were as follows: (1) working as a lifestyle coach, (2) being self-employed and/or affiliated to an organization, rehabilitation center, hospital, or municipality in the Netherlands, and (3) having been delivering lifestyle coaching to PLWDs in the Netherlands in the past 12 months. Inclusion criteria for implementation experts were as follows: (1) being self-employed and/or affiliated to an organization, rehabilitation center, hospital, or municipality in the Netherlands, and (2) having experience of implementing one or more lifestyle coaching programs for PLWDs in the Netherlands in the past 12 months. Lifestyle coaching was defined here as any type of behavioral support (conversation) between a lifestyle coach and a client on starting, changing, and/or maintaining a healthy lifestyle. Lifestyle coaching includes one or multiple conversations about one of the following lifestyle factors: nutrition, sleep, physical activity, stress management, or positive social connections.

Recruitment of professionals was carried out via the authors’ and Stichting Special Heroes’ network. Professionals were initially contacted via email by one of the co-authors (MF, LV, and TH) and asked whether they were interested in participating in the study. After showing interest in participating, potential participants received more detailed information about the study via email from the first author (EHD). The same email contained a link to a short online questionnaire (Qualtrics, Provo, UT, USA) to screen potential participants regarding the inclusion criteria. If participants met the inclusion criteria and digitally signed the consent form, they were forwarded an additional set of questions on their background, expertise, work setting, and experience with implementation and/or delivering lifestyle coaching for PLWDs. After completing the questionnaire, participants were contacted by the first author (EHD) to schedule an appointment for the interview.

### 2.3. Data Collection

Before the start of the interview, participants were informed about the reason for performing the study and the duration, structure, voluntary basis, and anonymity of the interview. Additionally, researcher EHD, who conducted the interviews, introduced herself and researcher JN, who was passively (i.e., with camera and microphone turned off) present during the interviews, taking field notes and discussing the interview directly afterwards. After ensuring that everything was clear and that there were no more questions, the recording started. The interviews were conducted via Microsoft Teams (Microsoft, 2022) or telephone. Recordings of the interviews were made with the use of the recording function within Microsoft Teams. The subtitling function created a first draft for the transcripts of the interviews. The recordings and subtitles were saved anonymously and stored internally, secured by passwords. They were removed after finalizing the study and data were not shared with anyone but the research team.

The first author (EHD) identified topics for the interview guide and drafted the first version of the guide based on discussions with other team members, inspired by the key domains and constructs of the Consolidated Framework for Implementation Research (CFIR) [19,20]. The CFIR outlines five domains that help to understand the implementation of interventions or programs: Intervention Characteristics, Inner Setting, Outer Setting, Characteristics of Individuals, and Process. Within Intervention Characteristics, a distinction is made between core components (i.e., the essential elements of the intervention) and the adaptable periphery (i.e., elements that can be tailored to the specific organizational setting). An example of a core component of the Healthy Habits Coaching is the telephone-based delivery model and the involvement of volunteer coaches. The lifestyle domains addressed during coaching sessions and the number of sessions offered are examples of adaptable elements. Since the Inner Setting mainly relates to elements within XX, there were no questions related to this domain in the interview guide. The Outer Setting for Healthy Habits Coaching is the setting that the interviewed professionals are familiar with, and comprises external policies and incentives and existing networks between XX and clinical and community organizations. Regarding Characteristics of Individuals, questions about beliefs and knowledge of Healthy Habits Coaching were added to the interview guide. Lastly, the participants were asked questions related to the Process of implementing interventions, in particular about planning and engagement of key individuals.

Co-authors (JN, MF, LV, TH, and FH) reviewed the interview guide and provided various rounds of feedback. By engaging our non-academic team members (ML and LV) in the development process, we ensured that questions were relevant and useful to them. The interview guide was finalized after a pilot session with a lifestyle coach. Participants were asked about their perceptions of Healthy Habits Coaching in general and its specific aspects (Table 1) with the use of questions like *What is your first impression of Healthy Habits Coaching?*, and *How do you compare Healthy Habits Coaching to other lifestyle coaching programs that you are familiar with, with regard to free participation?* Supplementary File S1 contains an infographic that illustrates the Healthy Habits Coaching program, as discussed during the interviews. Supplementary File S2 includes the final interview guide in Dutch.

### 2.4. Data Analysis

Each interview was transcribed verbatim. The interviews were primarily analyzed by researcher EHD using ATLAS.ti (version 22.0.7, Scientific Software Development GmbH) following a directed content analysis [21] focusing on the core domains of the CFIR (i.e., innovation, individuals, setting, and process). First, a codebook was created by co-authors EHD and JN after coding two transcripts. The codebook included open codes related to the core CFIR domains: innovation, individual, setting, and process. The first author (EHD) then coded the remaining transcripts. Minor changes to the codebook were made throughout the coding process. After coding all transcripts, EHD identified key topics within each core domain after several discussions with co-authors (JN, TH, and FH). The first author (EHD) wrote a detailed description of each topic. Descriptions were finalized after input and reflections from all co-authors (JN, TH, MF, LV, and FH), who acted as ‘critical friends’ [22]. Quotes from the interviews were selected to illustrate the findings. Quotes were translated into English and, if needed, readability was enhanced by making editorial changes. The resulting quotes were sent to the participants to ask for confirmation for accuracy of the translation and for consent to include them in the paper. Supplementary File S3 shows the original (Dutch) quotes. The participants were given pseudonyms to enhance readability.

## 3. Results

### 3.1. Participants

Fourteen lifestyle coaches were approached with a request to participate in the study. Eleven of them replied; three did not wish to participate due to lack of time; and three others did not meet the inclusion criteria. Of the included lifestyle coaches, 80% had completed a lifestyle coach education program, 20% were accredited lifestyle coaches, 40% were coaches focusing on physical activity and sport, and 60% also incorporated other domains of lifestyle in their coaching sessions. Seven implementation experts were approached; six of them replied, of whom one did not meet the inclusion criteria. Of the included implementation experts, 60% worked in a clinical setting (hospital or rehabilitation center).

Among the included participants (n = 10), 70% identified as women and 30% as men, with a mean age of 47.1 years (±11). Regarding their sexual orientation, 60% identified as heterosexual, and 10% as asexual; 10% identified as unknown, and 20% did not wish to answer the question. All participants were born in the Netherlands. Participants’ characteristics are presented in Table 2. The interviews ranged from 26 to 54 min, with a mean of 38 min. Interviews were conducted via Microsoft Teams (n = 9) or via telephone (n = 1).

### 3.2. Perceptions and Thoughts on Healthy Habits Coaching (Innovation and Individuals)

#### 3.2.1. Relative Advantage

Several participants explained that it is more difficult to achieve and sustain a healthy lifestyle for PLWDs compared to those without a disability and, hence, that a lifestyle coaching program like Healthy Habits Coaching could be valuable for PLWDs.

Because your whole life is just more difficult [for PLWD]. So, also going to the store to buy healthy products or performing moderate to intensive physical activity for 30 min a day is a lot more difficult. (…) So, yes, a bit of assistance with that is valuable, I think. (Mark, implementation expert)

The vast majority of the participants were positive about the program and indicated that it could be a valuable addition to existing lifestyle coaching programs currently available for PWLDs in the Netherlands. In line with that, David and Jennifer underlined the importance of the availability of different lifestyle coaching programs for PLWDs, as not everyone benefits from the same type of support. On the other hand, Linda, Emily, and Paul considered Healthy Habits Coaching to be comparable to the lifestyle coaching programs that were already offered to PLWDs in the physical activity counseling centers. Of note, many rehabilitation centers and hospitals in the Netherlands have a physical activity counseling center in which patients are offered tailored counseling on achieving and/or maintaining an active lifestyle after rehabilitation [23]. For example, Paul (lifestyle coach) mentioned the following: ‘Because essentially, we also do Get in Motion, but we do it with professionals and we do it based on subsidy funds.’

#### 3.2.2. Healthy Habits Coaching Components

Participants had different perceptions and opinions on Healthy Habits Coaching components. Hence, they suggested a broad range of suitable target populations for the program, as well as a broad range of populations for whom Healthy Habits Coaching would be less suitable. Overall, participants extensively underlined the need for flexibility in a coaching program, for instance adjusting a program’s mode of delivery (in-person or online) and type of coaching sessions (one-on-one or group coaching) based on the needs and wishes of a client.

Regarding the free coaching service (no cost for participation in Healthy Habits Coaching), participants noted both positive and negative aspects. Only three participants were positive about the free participation in Healthy Habits Coaching; they stated that it enhances its accessibility, especially for those clients with a low income. Conversely, Lisa reasoned that it might decrease a client’s motivation and commitment to the program, since there are no financial consequences of, for instance, not showing up. Furthermore, three participants noted that clients might still have to pay for actually performing the healthy behavior, for instance when buying a gym membership. They explained that it is therefore particularly important to focus on modifying a client’s stage of change towards a healthier lifestyle during a lifestyle program, so that they are more likely to be willing to pay to maintain their healthy behavior after finishing the program.

It is always easier to join something if it is for free than when you have to pay for it. And maybe you pay if you want a second round of the program. But then you also know what to expect and you might have a positive experience. So, then you can do a better cost-benefit analysis to decide whether you want to keep doing it. (Michelle, implementation expert)

Eight participants raised questions regarding the use of volunteers as coaches. That is because, according to them, lifestyle coaches require sufficient knowledge of a client’s condition, the targeted lifestyle domain and practical tools on how to gain a certain behavior, skills like conversation techniques, and personal characteristics like empathy. They questioned whether volunteers could develop these skills and knowledge through training. David and Paul suggested screening volunteers for a predefined set of skills and knowledge before appointing them as coaches within Healthy Habits Coaching.

You rather ensure that the volunteers are well equipped. (…) So, I could imagine that you create a profile for volunteers that contains several of the aspects that we discussed earlier, at least basic, and that a volunteer should meet that profile. (David, implementation expert)

Regarding coaches with lived experience, four participants explained a pitfall specifically for coaches with lived experience: they might be too focused on their own experience and consequently project it onto the client. On the other hand, six participants underlined that having lived experience as a coach might have a positive effect on the coaching, since these coaches can more easily show empathy and understand certain aspects of a disability, enhancing the creation of a relationship of trust with the client. In addition, they can serve as a role model for their clients.

### 3.3. Perceptions on Where and How to Implement Healthy Habits Coaching (Setting and Process)

#### 3.3.1. Compatibility of Healthy Habits Coaching

Participants discussed Healthy Habits Coaching’s compatibility with existing programs and processes, for instance with rehabilitation programs. During or soon after discharge was suggested as a suitable moment to refer clients to Healthy Habits Coaching, as their motivation to obtain and/or maintain a healthy lifestyle is likely high due to their positive experiences during rehabilitation. ‘The part of physical activity is really a major part of rehabilitation care and people experience during rehabilitation what it means to their body and consequentially, they are often really motivated to maintain it’ (Susan, lifestyle coach).

On the other hand, participants mentioned that a client might not be ready to change their behavior until after finishing rehabilitation and resettling their daily lives. In this case, it may be better to ‘plant a seed’ and wait before presenting a coaching program until the client is open for change.

Preferably we would do it within rehabilitation, but you see that during rehabilitation, some people are not open for it yet. (…) So, you could do something, but what is of importance is that you first plant a seed, simply with knowledge and the like, and thereafter, after you did that, that people themselves return with the fact that they are open to do it. (Emily, implementation expert)

For Healthy Habits Coaching specifically, Emily suggested that the program could be used as a low-key stepping stone into other (paid) lifestyle coaching programs. Nancy and Linda further suggested that clients could be redirected to Healthy Habits Coaching if they have to wait for regular coaching, or if they are not ‘ready’ yet after finishing the regular coaching period that is offered as part of their rehabilitation treatment. Indeed, several participants experienced that the success of lifestyle coaching mainly depends on a client’s motivation, sense of personal responsibility, and engagement. A client should thus be referred to a coaching program whenever they are ready.

#### 3.3.2. Where to Implement Healthy Habits Coaching

Almost all participants shared ideas about where to implement Healthy Habits Coaching. Five of them suggested implementing it at national level, so that all lifestyle coaches working with PLWDs could refer their clients to Healthy Habits Coaching, and thus that clients from anywhere in the Netherlands can be referred to the program.

For the lifestyle counseling center in our hospital we are always looking for such programs to refer someone to. So, theoretically, if this program exists, we could send our patients to Healthy Habits Coaching. Knowing that there is a focus on physical activity, nutrition and sleep. And that coaches with lived experiences are doing the conversations. (Michelle, implementation expert)

Participants noted that if the program were to be implemented at a national level there should be enough coaches available, and these coaches should be matched with clients by the program’s owner. Paul expressed that it would be better to organize it on a local community level, for example via municipalities, since that would be more manageable. Emily, David, and Jennifer suggested another option: offering Healthy Habits Coaching in a local clinical setting, for example within a hospital or rehabilitation center. Michelle and Paul emphasized that regardless of where Healthy Habits Coaching would be implemented, it should be easy for different professionals (e.g., doctors, therapists, coaches, etc.) to refer people to the program.

#### 3.3.3. How to Implement Healthy Habits Coaching

Mark elaborated on the importance of building enthusiasm within an organization regarding the implementation of a lifestyle program. According to him, enthusiasm is enhanced if the implementation process is clear, relatively easy, and if the program itself has added value. In line with that, Paul noted that an organization’s ‘need’ for Healthy Habits Coaching should be assessed if the program might be implemented in that setting, so that it is implemented there where it adds something. Also, Paul stated that a promotion campaign for Healthy Habits Coaching could help to make the program known to all the organizations and/or municipalities that could potentially implement it and in all locations where the target population can be found, and thus, from where they could be referred to the program. Several participants underlined that promotion material should be concise and clear, with the use of accurate pictures and vocabulary. Mark suggested adding a clear explanation on why both coaches and clients should participate in Healthy Habits Coaching.

Yes, for the target group ‘well, why is it important that you have a healthy lifestyle?’, short. And secondly what the final result will be. (…) And for the coach more like ‘what does it bring me as a coach’, or ‘why is it important that I sign up for this’. (Mark, implementation expert)

Additionally, Emily suggested creating a website to refer to for additional practical information as well as information on the program’s underlying mechanisms and (financial) responsibilities. The need for transparency and clarity about the latter was also clearly underlined by Jennifer (lifestyle coach) ‘… else it seeps away and then the conclusion is ‘yes, there wasn’t that much interest for it’. But actually, the basis underneath wasn’t accurate; the organizational and financial basis was too difficult.’

Mark and Michelle stated that it is important to engage all involved parties and, in particular, decision-makers as soon as possible. Susan noted that decisions on the implementation of new programs are made higher up, that is by managers or board members. Lisa, Paul, and Michelle experienced the essence of engagement of those decision-makers.

Yes, well, in the end the managers determine how much money we will get, whether I get to stay working here, whether I can do my job. (…) So, we have to make sure to show: ‘well, sport is important for people with disabilities. They want it. Give us the chance, give us the money, give us the accommodation, give us the colleagues, etcetera.’ (Paul, implementation expert)

## 4. Discussion

We conducted semi-structured interviews with lifestyle coaches and implementation experts to explore their experiences and perceptions regarding lifestyle coaching for PLWDs and their perceptions of the implementation of Healthy Habits Coaching. Grounded in practical, real-world issues encountered by Stichting Special Heroes (a Dutch community organization), this study focused on the preparation phase of adapting a lifestyle program for a new context [11] and, ultimately, contributing to enhancing well-being among PLWDs by promoting healthy lifestyle behaviors. Aligning with the CFIR domains, our findings provide directions for how, where, and by whom the Healthy Habits Coaching program could be prepared for implementation in Dutch rehabilitation and/or community settings. Participants discussed their perceptions and thoughts related to the innovation (of Healthy Habits Coaching), to individuals (counselors), to the setting (rehabilitation and community settings), and to the process (promotion and implementation strategies).

### 4.1. The Innovation and Involved Individuals

While participants were generally positive about Healthy Habits Coaching and talked about the importance of offering counseling support to PLWDs, they had mixed views and perceptions on two key components of Healthy Habits Coaching. The first component was the free coaching service. A remarkable finding was that a free coaching service was not necessarily perceived as positive by all participants. Participants mentioned that a free service could lower a client’s commitment to that service, and thus to change their behavior. This is in line with the literature on the effects of incentives on behavior change [24,25,26], describing its complexity and sometimes rather paradoxical effects, especially in the short-term. A systematic review and meta-analysis of Giles et al. [24] concludes that including financial incentives or penalties in behavior change programs could enhance attendance rates and programs’ effectiveness. On the other hand, a free coaching service was perceived as more accessible and inclusive, in particular for those with a lower income who have a higher risk of developing unhealthy lifestyle behavior. To enhance adherence to a behavior change program like Healthy Habits Coaching, a possible solution could be to request a fee for no-show to counseling sessions [26,27], while still maintaining its free participation component. Another possible strategy to balance accessibility with participant commitment is to offer the initial portion of the program for free, and then introduce a fee for continued or enhanced support for those who want to engage further.

The second component was the use of volunteers with lived experience as coaches. Some participants raised concerns related to professional skills and personal characteristics when using volunteers as coaches. Indeed, a systematic review on factors enhancing physical activity participation in PLWDs underlined the need for instructors with multi-faceted knowledge [28]. It should thus be ensured that volunteers have a sufficient set of skills and knowledge, for instance by using a pre-selection process or by providing training, before appointing them as coaches within Healthy Habits Coaching. On the other hand, previous studies on the efficacy of peer health coaching interventions in people with disabilities have shown promising findings in terms of behavior change outcomes [29,30]. In the Netherlands, most lifestyle programs for PWLDs are delivered by professional coaches who typically do not have lived experience. While people with lived experience do work in rehabilitation centres to support rehabilitation clients, their roles are not specifically focused on promoting healthy lifestyle behaviors, and they often lack formal training in this area. Healthy Habits could fill this gap by offering a unique model in which coaching is delivered by trained and skilled people with lived experience. In cases where there are not enough coaches with lived experience available, and if funding permits, a hybrid model could be considered, allowing the service to be delivered by both volunteer coaches with lived experience and professional coaches.

While participants had mixed views and perceptions on certain aspects of Healthy Habits Coaching, they agreed on the importance of tailoring lifestyle coaching to clients’ preferences and needs. This is in line with a recent article of [31] describing experiences and opinions of PLWDs participating in health interventions and lifestyle coaches. Other studies align with these findings, reflected by enthusiasm of both engaged professionals and clients regarding to tailoring lifestyle counseling programs [23,32,33,34].

In sum, tailoring of a lifestyle coaching program is possible through the components in the adaptable periphery: they can be adapted to either opportunities in a certain setting or situation (i.e., the availability of coaches with lived experience) or the client (i.e., the number of coaching sessions that are required to meet their needs. Future research is needed to provide tangible strategies to optimally tailor the coaching program to the specific setting and needs of the end users.

### 4.2. Implementation Setting and Process

Participants provided a variety of suggestions on where and how Healthy Habits Coaching could be implemented. Regarding the setting, participants provided suggestions to implement the program in rehabilitation centers, as rehabilitation is an ideal moment to promote healthy lifestyle behaviors. The Netherlands is unique in the way that physical activity promotion activities, including counseling support, have been implemented in rehabilitation and hospital settings across the country to support PLWDs to become and stay physically active after rehabilitation [23]. Participants also mentioned that some clients may not feel ready to receive lifestyle counseling during or after their rehabilitation. The implementation of Healthy Habits Coaching could supplement the existing lifestyle programs implemented in the Dutch rehabilitation system by providing more flexibility, as clients can start the counseling program when they feel ready. Participants also suggested involving municipalities in the implementation of the Healthy Habits Coaching program. In the Netherlands, municipalities have important roles and responsibilities to promote a healthy lifestyle among the Dutch population. For example, municipalities offer various lifestyle coaching services for people with and without disabilities (e.g., community sport coaches [35,36]. Healthy Habits Coaching could add to the existing lifestyle programs by focusing specifically on PLWDs living in the community. As reaching PLWDs via community-based programs can be challenging, future research should focus on how to optimally reach a diverse group of PLWDs to engage in a community-based lifestyle coaching program.

Regarding the implementation process, participants suggested preparing a campaign to promote the Healthy Habits Coaching program and create enthusiasm and awareness for the implementation of the program. Aligning with Roger’s Diffusion of Innovations Theory, the use of champions may be an effective way to promote the program among rehabilitation professionals [37]. Participants emphasized that promotion activities should target decision-makers (e.g., managers and board members), which aligns with the previous literature on drivers for successful implementation of integrated care [38]. Future research is needed to systematically identify barriers and facilitators in implementing the Healthy Habits Coaching program in specific settings and to map implementation strategies to overcome these barriers and leverage facilitators, using implementation science tools and frameworks, such as the CFIR-ERIC-tool [39].

### 4.3. Scientific and Practical Implications

This study adds to the implementation science and behavioral medicine literature by illustrating how an existing, evidence-based lifestyle program (i.e., GiM) can be adapted and prepared for implementation in a new setting. Systematically exploring in-depth perceptions and opinions from a diverse group of professionals working in the area of lifestyle coaching for PLWDs prior to implementation will enhance program acceptability. We provide an example of how existing knowledge and best practices on lifestyle coaching for PLWDs can be used in a new setting (e.g., non-English countries). The study adds to the healthy behavior change literature by providing new insights into perceptions of a diverse group of professionals on offering, delivering, and/or implementing lifestyle coaching for PLWDs in both rehabilitation and community settings.

Our findings provide directions for how, where, and by whom the Healthy Habits Coaching program could be prepared for implementation in Dutch rehabilitation and/or community settings. Based on the findings of this study and the existing literature, our research team formulated suggestions for the implementation of Healthy Habits Coaching, which are summarized in Table 3. While these are preliminary suggestions for implementation, it is important that individual coaching sessions are tailored to the specific needs and preferences of each client; this includes the focus of the coaching (e.g., lifestyle behaviors), the mode of delivery (e.g., phone or virtual), and the duration of the coaching program. Future research is needed to further refine these suggestions and to better understand optimal referral pathways and delivery strategies for the Healthy Habits Coaching program. Furthermore, additional research is needed to better understand the barriers and facilitators of long-term sustainability of the Healthy Habits Coaching program, including factors such as funding, ongoing training and education, user resistance, and continuous evaluation. Developing a model in which the program is owned and led by the organization responsible for its coordination and delivery may support sustainability. Ongoing training and quality improvement efforts could be strengthened through partnerships between researchers, community organizations, PLWDs, and lifestyle coaches. Such partnerships may also help to foster acceptability and can optimize support and promote the sustainable integration of the program into existing systems. These directions are relevant for Stichting Special Heroes and other organizations, as well as professionals involved in implementing the Healthy Habits Coaching. Furthermore, the findings of this study can be used by other organizations and professionals to inform the adaption and/or implementation of lifestyle coaching for PLWDs in their own settings.

### 4.4. Strengths and Limitations

This study’s main strength lies in the use of an IKT approach (i.e., the research was conducted in collaboration with the organization that adapted and will execute Healthy Habits Coaching) and a pragmatic perspective as its basis. During the interviews, professionals had to ground their perceptions on a hypothetical version of Healthy Habits Coaching, as the program was not fully developed at the start of this study. This gave Stichting Special Heroes the opportunity to incorporate the results in the further development of Healthy Habits Coaching. In fact, the Healthy Habits Coaching program has been launched and is currently available to all PLWDs in the Netherlands [40]; coaches receive training before and between sessions during delivery of coaching, and Healthy Habits Coaching is offered as an option to be referred to via the existing lifestyle counseling centers within rehabilitation centers and a national website (www.beterverwijs.nl). This illustrates the usability of the knowledge that was the result of this study [16].

The study has some limitations. First, despite including participants with a wide variety of professional backgrounds, the majority of participants identified as women and heterosexual. Particularly, all interviewed lifestyle coaches were women, which may have introduced gender bias and limited the perspectives captured, particularly regarding how male coaches might approach or experience similar issues. In line with this, the study was conducted in a Dutch setting. The findings are therefore highly relevant for the Dutch context, but they might be less transferrable to other countries. We underscore the importance of conducting similar contextual studies in diverse settings to account for the unique environmental, social, and institutional factors that shape PLWDs’, coaches’, and professionals’ (lived) experience. However, to assess the broader applicability of findings and to capture the diversity of experience, it is equally important to replicate such studies across different contexts. Another limitation is that participants were recruited through purposive sampling, mainly via the authors’ networks. Although not all participants were (directly) connected to our network and we obtained mixed findings related to the feelings and opinions about Healthy Habits Coaching, recruitment bias could have played a small role. Furthermore, we interviewed implementation experts based on self-reported expertise. Participants were not necessarily implementation researchers, but rather individuals having practical experience with implementing lifestyle programs for PLWDs. Another limitation is that this study did not include lifestyle coaches with lived experience. However, their perceptions with regard to Healthy Habits Coaching would be valuable as they play a key role in the program. Future studies should aim to specifically include people with lived experience with an interest in and/or experience with lifestyle coaching to PLWDs.

## 5. Conclusions

The findings of this study provide directions for how, where, and by whom an adapted lifestyle program (Healthy Habits Coaching) for PLWDs could be implemented in Dutch rehabilitation and community settings. This study demonstrates an example of how an evidence-based lifestyle program can be prepared for implementation in a new setting, presenting an efficient and promising strategy to enhance overall well-being among PLWDs.

## Figures and Tables

**Table 1:** Healthy Habits Coaching compared to Get in Motion.

	Get in Motion	Healthy Habits Coaching	Key Modifications
**Mode of delivery**	Telephone	Online or telephone	Yes
**Costs for participation**	Free	Free	No
**Approach**	One-on-one	One-on-one	No
**Counseling technique**	Brief Action Planning	Brief Action Planning	No
**Coaches**	Volunteers	Volunteers, preferably coaches with lived experience	Yes
**Number of coaching sessions**	Not specified	6–8 within a 3–6 month period	Yes
**Targeted lifestyle domains**	Physical activity	Physical activity, nutrition, sleep	Yes
**Target population**	People with physical disabilities	People with physical disabilities and/or chronic diseases	Yes

Note. This comparison made based on the Get in Motion program that was re-launched at the start of the pandemic (April 2020).

**Table 2:** Characteristics of participants (n = 10).

Participant’s Pseudonym	Group of Professionals	Function	Work Setting	Age (Years)	Gender
Jennifer	Lifestyle coach	Physical activity counselor	Rehabilitation center 1	48	Woman
Linda	Lifestyle coach	Physical activity counselor	Rehabilitation center 1	-	Woman
Susan	Lifestyle coach	Physical activity therapist	Rehabilitation center 2	41	Woman
Lisa	Lifestyle coach	Lifestyle and vitality coach	Self-employed	50	Woman
Nancy	Lifestyle coach	Sports and lifestyle coach	Rehabilitation center 6	30	Woman
David	Implementation expert	Rehabilitation physician	Hospital 1	61	Man
Emily	Implementation expert	Manager of department of rehabilitation and sports	Rehabilitation center 4	51	Woman
Paul	Implementation expert	Sports counselor, manager sports counseling center	Rehabilitation center 8	61	Man
Michelle	Implementation expert	Implementation scientist	Hospital 2	37	Woman
Mark	Implementation expert	Teacher, researcher	Applied university 1	33	Man

**Table 3:** Suggestions for the adoption and implementation of Healthy Habits Coaching.

Components	Suggestions for the Implementation of Healthy Habits Coaching
Innovation (what)	- To successfully implement the Healthy Habits Coaching, it is important that the added value and the scientific foundation of the program are clearly communicated. It may help to outline the unique characteristics of Healthy Habits Coaching compared to other existing lifestyle promotion programs.
Individuals (who)	- Coaches should have sufficient skills and knowledge to deliver lifestyle coaching. They should be trained in coaching skills (Brief Action Planning) before being assigned as a coach. Trained coaches with lived experience are preferred. - People with a variety of physical disability and/or chronic diseases could be referred to the program and benefit from lifestyle coaching.
Setting (where)	- Healthy Habits Coaching can co-exist with the lifestyle programs (e.g., Physical Activity Sports Centers) implemented in Dutch rehabilitation care. Rehabilitation professionals can then refer their clients to Healthy Habits Coaching as an additional lifestyle support program available after their rehabilitation treatment. - Healthy Habits Coaching can also be operated at national level to allow implementation of the program in community settings. For example, clients could sign up themselves or be referred by professionals working in community organizations and services
Process (how)	- The implementation of Healthy Habits Coaching should be coordinated by Stichting Special Heroes. Engagement of various stakeholders early and throughout the implementation process is essential. The implementation could start at local level via rehabilitation centers interested in referring clients to Healthy Habits Coaching. - A promotion campaign focusing on explaining the (scientific) foundation and added value of Healthy Habits Coaching may help the adoption process in various rehabilitation and community settings.

## Data Availability

De-identified data, including survey responses and transcripts of interviews, from this study are not available in a public archive to protect participants’ confidentiality De-identified survey data from this study may be made available (as allowable according to institutional IRB standards) upon reasonable request by emailing the corresponding author.

## Supplementary Materials

The following supporting information can be downloaded at https://www.mdpi.com/article/10.3390/healthcare13161978/s1. Supplementary File S1. Infographic: Healthy Habits Coaching (Dutch). Supplementary File S2. Interview Guide (Dutch). Supplementary File S3. Quotes (original (Dutch) and translations).
