# Supplementary material for: Professionals’ Perceptions on Implementing an Adapted Lifestyle Coaching Program for People with Physical Disabilities
Source: Healthcare (Basel). 2025 Aug 12;13(16):1978. doi: 10.3390/healthcare13161978 (PMC12385834; doi:10.3390/healthcare13161978)
Supplement: Supplementary file 1 [file healthcare-13-01978-s001.zip › healthcare-3730290-supplementary.pdf]

Supplementary File S1. Infographic Healthy Habits Coaching (Dutch)

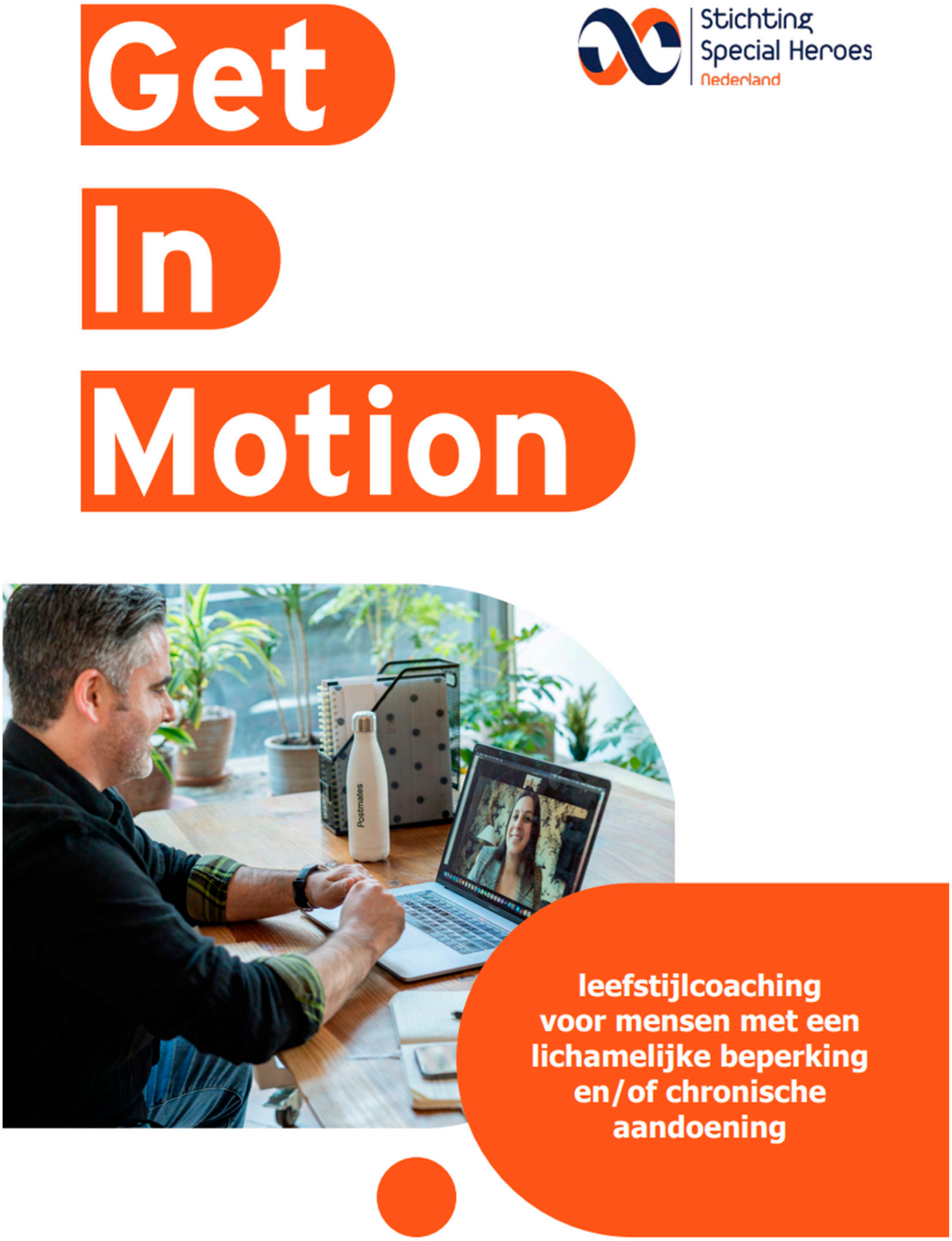

Wat is Get In Motion?

Get In Motion is een gratis online/telefonische leefstijl-coachingservice voor mensen met een lichamelijke beperking en/of chronische aandoening.

Leefstijlcoaching

- De insteek is laagdrempelig en gericht op stappen maken in gedragsverandering op gebied van bewegen, voeding of slapen.
- Zes tot acht individuele gesprekken verspreidt over drie tot zes maanden.

De service

- De coaches van Get In Motion zijn getrainde (ervaringsdeskundige) vrijwilligers.
- Deelname aan Get In Motion is kostenloos.
- Persoonlijke coaching op basis van behoeften van de deelnemer.

Inschrijven

- Zowel coaches als deelnemers kunnen zich aanmelden voor Get In Motion via Stichting Special Heroes Nederland
- Dit kan door een mail te sturen naar: GIM@specialheroes.nl

Supplementary File S2. Interview guides (Dutch)

At the time of the interviews, the new program was still called Get in Motion (instead of Healthy Habits Coaching). Therefore, Get in Motion or GiM in the interview guides can be read as Healthy Habits Coaching.

Lifestyle coaches

Introductie

Nogmaals bedankt dat u wilt deelnemen aan dit onderzoek.

Ik zal mij eerst even voorstellen. Ik ben XX, ik ben 24 jaar en studeer momenteel de master gezondheidswetenschappen aan de Vrije Universiteit in Amsterdam. Voor deze masteropleiding ben ik dit halfjaar bezig met mijn afstudeer stage, waarvoor ik dit onderzoek uitvoer.

In deze studie zijn we benieuwd naar ervaringen met betrekking tot het aanbieden, geven of implementeren van leefstijl coaching voor mensen met een motorische beperking en/of chronische aandoening in Nederland. Hiervoor interviewen we mensen die hier ervaring mee hebben, en werkzaam zijn in verschillende settings. Zo hopen we een breed beeld te krijgen van wat factoren zijn die de uitrol van leefstijl coaching kunnen beïnvloeden. In het bijzonder willen we onderzoeken wat de opvattingen zijn van deze ervaren professionals met betrekking op Get in Motion, een nieuw leefstijl coaching programma voor mensen in deze doelgroep. De studie wordt gedaan in samenwerking met Stichting Special Heroes.

Voordat we deze afspraak gemaakt hebben, heeft u een online vragenlijst ingevuld met een aantal vragen. Tevens heeft u aangegeven toestemming te geven voor deelname aan dit onderzoek. Heeft u daar nog vragen of onduidelijkheden over?

Het interview zal naar verwachting ongeveer 30 minuten duren. Jesse is ook in deze meeting aanwezig. Hij is betrokken bij hetzelfde onderzoek, maar zal in deze meeting enkel op de achtergrond aanwezig zijn, en mij achteraf feedback geven.

Graag zou ik dit interview opnemen. Deze opname wordt anoniem opgeslagen en op een interne, beveiligde locatie van de Vrije Universiteit Amsterdam. De opname is bedoeld zodat ik deze achteraf kan terug luisteren en het interview kan uitwerken. Gaat u daarmee akkoord? Dan zet ik nu de opname aan.

Deel 1 – opening

1. Kunt u mij iets vertellen over uw huidige functie?
    a. Hoe lang doet u dit al?
    b. Hoe bent u hier terecht gekomen?
2. Kunt u mij iets vertellen over uw ervaring als leefstijl coach voor mensen met een motorische beperking en/of chronische aandoening?
    a. Als u terugdenkt aan het begin van uw carrière als leefstijl coach: is uw werk veranderd over de jaren?
    a. [U heeft een vragenlijst ingevuld waarin u aangaf dat u een motorische beperking en/of chronische aandoening heeft. Nu bent u als coach actief [geweest] bij [leefstijl coaching programma / werk context]. U bent dus een soort ervaringsdeskundige. Kunt u iets vertellen over wat voor meerwaarde dat heeft in uw positie als coach?]
3. Wat vindt u van leefstijl coaching [voor mensen met een motorische beperking en/of chronische aandoening]?
4. Wat zijn voor u essentiële kenmerken van goede leefstijl coaching?
5. Wat is voor u belangrijk voor het opbouwen van het contact met de cliënt?
    a. Hoe ging/gaat het opbouwen van dit contact?
        i. Doorvraag: vraag naar vertrouwensband

Deel 2 – perceptie over GiM

In de mail die u van mij ontvangen heeft, zat een infographic in de bijlage. Ik zet hem op het scherm. Dit gaat over Get in Motion, een nieuw leefstijl coaching programma voor mensen met een lichamelijke beperking. Het programma wordt aangeboden door Stichting Special Heroes. Heeft u naar de infographic kunnen kijken (ja / nee)?

6. Wat is uw eerste indruk van Get in Motion?
    a. [U gaf nu aan dat u de flyer … vindt. Wat vindt u van de inhoud van het programma?]
    b. [Wat spreekt u aan in GiM?]
    c. [Wat vindt u minder goed aan GiM?]
    d. Zijn er dingen die u in het bijzonder opvielen?
7. Hoe ziet u GiM ten opzichte van andere leefstijl coaching programma's waarbij u betrokken bent [geweest]? Wat zijn verschillen tussen GiM en andere leefstijl coaching programma's waarbij u betrokken bent?
    a. Gratis voor deelnemers
    b. Online/telefonisch
    c. 6-8 gesprekken over een periode van 3-6 maanden
    d. Coaches: getrainde (ervaringsdeskundige) vrijwilligers
    e. Focus op een vraag die door de cliënt is aangeleverd

Deel 3 – perceptie over de uitrol van GiM

8. Hoe zou GiM in [huidige werk context] passen?
    a. Zou u dat nader willen toelichten?
    b. [Wat zijn factoren die dat beïnvloeden?]
    c. In wat voor setting ziet u GiM voor u?
    d. Hoe zou GiM aangepast kunnen worden zodat het in [huidige werk context] zou passen?

9. Zou het een aanvullende of vervangende service zijn?
    a. Voor wie?
    b. Waarom?
10. Zou u uw cliënten doorverwijzen naar Get in Motion (ja / nee)?
    a. Waarom wel / niet?
    b. Wie zou u doorverwijzen? En wie niet?
11. Zou u uw collega's wijzen op het bestaan van Get in Motion (ja / nee)?
    a. Waarom wel / niet?
    b. Wie zou u doorverwijzen? En wie niet?

Deel 4 – afsluiting

12. We zijn bij het einde van het interview aangekomen. Zijn er zaken die we nog niet besproken hebben en waar u nog iets over kwijt wil of is er nog iets waar u op terug wilt komen?
13. Heeft u interesse in het ontvangen van mijn verslag wanneer ik deze heb afgerond? Dat zal eind juni zijn.
    a. Wat is het mailadres waar ik het verslag tegen die tijd heen mag sturen?
14. Mag ik via de mail contact met u opnemen mocht ik eventuele aanvullende vragen hebben over dit interview?
15. [Een studiegenoot van mij,]Jesse, [die ook in deze meeting zit,] doet een soortgelijk onderzoek maar interviewt deelnemers van leefstijl coaching programma's. Kent u cliënten die u zou kunnen benaderen voor zijn onderzoek?

Dan stop ik nu de opname.

Implementation experts

Introductie

(same as for lifestyle coaches)

Deel 1 – opening

1. Kunt u mij iets vertellen over uw huidige functie?
    a. Hoe lang doet u dit al?
    b. Hoe bent u hier terecht gekomen?
2. Kunt u mij iets vertellen over uw ervaring met het [aanbieden / implementeren] van leefstijl coaching aan mensen met een motorische beperking en/of chronische aandoening?
    a. Als u terugdenkt aan het begin van uw carrière als […]: is uw ervaring met leefstijl coaching veranderd over de jaren?
3. Wat vindt u van leefstijl coaching [voor mensen met een motorische beperking en/of chronische aandoening]?
4. Wat zijn voor u essentiële kenmerken van goede leefstijl coaching?

Deel 2 – perceptie van GiM

In de mail die u van mij ontvangen heeft, zat een infographic in de bijlage. Ik zet hem op het scherm. Dit gaat over Get in Motion, een nieuw leefstijl coaching programma voor mensen met een lichamelijke beperking. Het programma wordt aangeboden door Stichting Special Heroes. Heeft u naar de infographic kunnen kijken (ja / nee)?

5. Wat is uw eerste indruk van Get in Motion?
    a. [U gaf nu aan dat u de flyer … vindt. Wat vindt u van de inhoud van het programma?]
    b. [Wat spreekt u aan in GiM?]
    c. [Wat vindt u minder goed aan GiM?]
    d. Zijn er dingen die u in het bijzonder opvielen?

6. Hoe vergelijkt u GiM met andere interventies waarbij u betrokken bent [geweest]? Hoe ziet u GiM ten opzichte van andere leefstijl coaching programma's waarbij u betrokken bent [geweest]?
    a. Gratis voor deelnemers
    b. Online/telefonisch
    c. 6-8 gesprekken over een periode van 3-6 maanden
    d. Coaches: getrainde (ervaringsdeskundige) vrijwilligers
    e. Focus op een vraag die door de cliënt is aangeleverd

Deel 3 – perceptie over de uitrol van GiM

7. Hoe zou GiM in [huidige werk context] passen?
    a. Zou u dat nader willen toelichten?
    b. Wat zijn factoren die dat beïnvloeden?
    c. In wat voor setting ziet u GiM voor u?
    d. Hoe zou GiM aangepast kunnen worden zodat implementatie in [huidige werk context] [mogelijk / aantrekkelijker / haalbaarder] is?
8. Zou het een aanvullende of vervangende service zijn?
    a. Voor wie?
    b. Waarom?
9. Zou u cliënten van [huidige werk context] doorverwijzen naar Get in Motion (ja / nee)?
    a. Waarom wel / niet?
    b. Wie zou u doorverwijzen? En wie niet?
10. Zou u uw collega's wijzen op het bestaan van Get in Motion (ja / nee)?
    a. Waarom wel / niet?
    b. Wie zou u doorverwijzen? En wie niet?

Deel 4 – afsluiting

11. We zijn bij het einde van het interview aangekomen. Zijn er zaken die we nog niet besproken hebben en waar u nog iets over kwijt wil of is er nog iets waar u op terug wilt komen?
12. Heeft u interesse in het ontvangen van mijn verslag wanneer ik deze heb afgerond? Dat zal eind juni zijn.
    a. Wat is het mailadres waar ik het verslag tegen die tijd heen mag sturen?
13. Mag ik via de mail contact met u opnemen mocht ik eventuele aanvullende vragen hebben over dit interview?
14. [Een studiegenoot van mij,]XX, [die ook in deze meeting zit,] doet een soortgelijk onderzoek maar interviewt deelnemers van leefstijl coaching programma's. Kent u cliënten die u zou kunnen benaderen voor zijn onderzoek?

Dan stop ik nu de opname.

Supplementary File S3. Quotes (original (Dutch) and translations)

Nr.	Quote (Dutch)	Translation
1	Want je hele leven is gewoon moeilijker [voor PLWD]. Dus dan is ook naar de winkel gaan om gezonde producten te kopen of 30 minuten per dag matig tot intensief bewegen is een stuk lastiger. […] Dus ja, een beetje begeleiding daarin is wel waardevol, denk ik.	Because your whole life is just more difficult [for PLWD]. So, also going to the store to buy healthy products or performing moderate to intensive physical activity for 30 minutes a day is a lot more difficult. (…) So, yes, a bit of assistance with that is valuable, I think.
2	Want bij vervanging dan overschrijf je iets wat al werkt, en ik denk dat toevoegen [van Healthy Habits Coaching] echt van meerwaarde is, omdat je dan voor specifieke doelgroepen dit kunt aanbieden en voor een ander weer iets anders.	Because replacement implies overwriting something that already works, and I think that adding it [Healthy Habits Coaching] is valuable, because then you can offer one thing to a specific target group and another thing to another group.
3	Want wij doen in principe Get in Motion doen wij ook, maar wij doen dat dus met professionals en wij leven dan wel van subsidiegelden.	Because essentially, we also do Get in Motion, but we do it with professionals and we do it based on subsidy funds.
4	Je hebt mensen die zijn wat zelfstandiger en die hebben alleen nog even dat laatste zetje nodig van waar kunnen ze precies terecht. Dus echt over het aanbod. Maar er zijn ook echt mensen die nooit hebben nagedacht over bewegen en die dan door een arts worden doorgestuurd.	There are people who are more independent, who only need the final push about where exactly they can go. So, it is actually more about the offer. But there are also people who have never thought about physical activity and who are then referred by a doctor.
5	De ene persoon vindt het heel prettig om in een groep leefstijl begeleiding te krijgen, omdat je dan met lotgenoten eigenlijk zit. En, nou ja, dat het misschien ook gezellig is en dat er dat groepselement aan zit. Maar de ander vindt het heel prettig om individuele leefstijl coaching te hebben en wil totaal niet met andere mensen hierover praten.	One person might prefer group coaching because you are surrounded with peers. And also, well, it might be fun because of the group element. But, someone else might prefer individual coaching and totally does not want to talk to other people about it.
6	Het is altijd makkelijker om ergens bij aan te sluiten als iets gratis is dan als je daarvoor moet betalen. En misschien als je een vervolg zou willen dat je dan wel betaalt. Maar dan weet je ook wat je kan verwachten en heb je misschien een positieve ervaring. Dus dan kan je beter die, die kosten-baten analyse afwegen of je het wel blijven doen.	It is always easier to join something if it is for free than when you have to pay for it. And maybe you pay if you want a second round of the program. But then you also know what to expect and you might have a positive experience. So, then you can do a better cost-benefit analysis to decide whether you want to keep doing it.
7	Als je er maar voor zorgt dat, dat, dat, dat die vrijwilligers ook goed geëquipeerd zijn. […] dus ik	You rather ensure that the volunteers are well equipped. (…) So, I could imagine that you create

	kan me voorstellen dat je van tevoren een soort profiel opstelt voor vrijwilligers waarin een aantal van die dingen die we eerder bespraken, in elk geval basaal, dat daaraan voldaan wordt	a profile for volunteers that contains several of the aspects that we discussed earlier, at least basic, and that a volunteer should meet that profile.
8	Het stukje bewegen is echt wel een groot deel van de revalidatiebehandeling en mensen ervaren tijdens de revalidatie wat het voor hun lijf betekent en zijn daardoor vaak echt wel gemotiveerd om het voort te zetten.	The part of physical activity is really a major part of rehabilitation care and people experience during rehabilitation what it means to their body and consequentially, they are often really motivated to maintain it.
9	…het liefst zouden we het doen binnen de revalidatie, maar je ziet dat in de revalidatie staat men er nog niet voor open. […] Dus dan zou je wel wat kunnen doen, maar wat dan van belang is dat je eerst een zaadje plant, gewoon met kennis en dergelijke, en dat als je dat dan hebt gedaan, dat de mensen zelf terugkomen met het feit dat ze open staan om het te doen.	Preferably we would do it within rehabilitation, but you see that during rehabilitation, some people are not open for it yet. (…) So, you could do something, but what is of importance is that you first plant a seed, simply with knowledge and the like, and thereafter, after you did that, that people themselves return with the fact that they are open to do it.
10	Vanuit het leefstijl loket in ons ziekenhuis zoeken wij natuurlijk naar zulke programma's waar we iemand heen kunnen sturen. Dus in theorie, als dit programma er is, dan zouden wij patiënten naar [Healthy Habits Coaching] kunnen sturen. Wetende dat patiënten daar bewegen, voeding en slaap aandacht voor is. En dat het inderdaad ervaringsdeskundigen zijn die dan in gesprek gaan.	For the lifestyle counseling center in our hospital we are always looking for such programs to refer someone to. So, theoretically, if this program exists, we could send our patients to Healthy Habits Coaching. Knowing that there is a focus on physical activity, nutrition and sleep. And that coaches with lived experiences are doing the conversations.
11	Maar ik lees nu hier dat het vrijwilligers worden. […] Want als je het gratis aanbiedt, dan kun je toch niet met professionals werken? […] Want als een professional bij ons op het centrum een taak erbij krijgt, dan gaat de baas altijd steigeren, van 'ja, maar daar hebben we geen ruimte voor, hebben geen geld voor'	But now I am reading here that they will be volunteers. (…) Because if you offer it for free, you cannot work with professionals, right? (…) Because if a professional in our center gets a new task, then the boss always directly objects, like 'yes, but we don't have space for that, we don't have money for it.
12	Maar uiteraard kun je het natuurlijk ook promoten bij alle artsen die er zijn, en die kunnen dan rechtstreeks ergens iemand heen doorsturen. […] Weet alleen dat er heel veel artsen zijn met wie je dan je programma moet uitdraaien. Ja, dat kost gewoon heel veel meer effort.	But of course you could also promote it amongst all doctors and they can directly redirect someone to something. (…) I simply know that there are a lot of doctors with whom you then have to roll-out your program. So, yes, that will just take a lot more effort.
13	Ja, voor de doelgroep echt van; 'nou ja, waarom is belangrijk dat je gezonde leefstijl hebt?', kort. En	Yes, for the target group 'well, why is it important that you have a healthy lifestyle?', short. And

	twee; 'wat het uiteindelijke resultaat is'. […] En dan voor de coach meer 'wat heb ik er als coach aan', of 'waarom is het belangrijk dat ik me hiervoor inschrijf'.	secondly what the final result will be. (…) And for the coach more like 'what does it bring me as a coach', or 'why is it important that I sign up for this'.
14	…anders sijpelt het weg en dan is de conclusie: 'ja, er was niet zoveel interesse voor'. Maar eigenlijk klopte die basis daaronder gewoon niet; het organisatorische en financiële plaatje was ingewikkeld.	… else it seeps away and then the conclusion is 'yes, there wasn't that much interest for it'. But actually the basis underneath wasn't accurate; the organizational and financial basis was too difficult.
15	Ja, nou ja, uiteindelijk bepalen die [ed: de leidinggevenden] natuurlijk hoeveel geld we krijgen, of ik mag blijven werken, of ik mijn werk kan doen. […] Dus we moeten zorgen dat we laten zien 'joh, sport is belangrijk voor mensen met beperking. Ze willen het graag. Geef ons de kans, geeft ons het geld, geven ons de accommodatie, geef ons de collega's et cetera'	Yes, well, in the end the managers determine how much money we will get, whether I get to stay working here, whether I can do my job. (…) So, we have to make sure to show: 'well, sport is important for people with disabilities. They want it. Give us the chance, give us the money, give us the accommodation, give us the colleagues, etcetera.
